# Short-term surgical outcomes of laparoscopy-assisted versus open D2 distal gastrectomy for locally advanced gastric cancer in North China: a multicenter randomized controlled trial

**DOI:** 10.1007/s00464-018-6391-x

**Published:** 2018-11-01

**Authors:** Zaozao Wang, Jiadi Xing, Jun Cai, Zhongtao Zhang, Fei Li, Nengwei Zhang, Jixiang Wu, Ming Cui, Ying Liu, Lei Chen, Hong Yang, Zhi Zheng, Xiaohui Wang, Chongchong Gao, Zhe Wang, Qing Fan, Yanlei Zhu, Shulin Ren, Chenghai Zhang, Maoxing Liu, Jiafu Ji, Xiangqian Su

**Affiliations:** 1 Department of Gastrointestinal Surgery IV, Key Laboratory of Carcinogenesis and Translational Research (Ministry of Education), Peking University Cancer Hospital & Institute, 52 Fucheng Road, Haidian District, Beijing, 100142 China; 2 Department of General Surgery, Beijing Key Laboratory of Cancer Invasion and Metastasis Research & National Clinical Research Center for Digestive Diseases, Beijing Friendship Hospital, Capital Medical University, Beijing, 100050 China; 3 Department of General Surgery, Xuanwu Hospital, Capital Medical University, Beijing, 100053 China; 4 Department of General Surgery, Peking University Ninth School of Clinical Medicine, Beijing Shijitan Hospital, Capital Medical University, Beijing, 100038 China; 5 Department of General Surgery, Beijing Tongren Hospital, Capital Medical University, Dongcheng, Beijing, 100730 China; 6 Department of Gastrointestinal Surgery, Key Laboratory of Carcinogenesis and Translational Research (Ministry of Education), Peking University Cancer Hospital & Institute, 52 Fucheng Road, Haidian District, Beijing, 100142 China

**Keywords:** Locally advanced gastric cancer, Laparoscopy-assisted distal gastrectomy, Open distal gastrectomy, Surgical outcomes, Clinical trial

## Abstract

**Background:**

Although laparoscopic surgery has been recommended as an optional therapy for patients with early gastric cancer, whether patients with locally advanced gastric cancer (AGC) could benefit from laparoscopy-assisted distal gastrectomy (LADG) with D2 lymphadenectomy remains elusive due to a lack of comprehensive clinical data. To evaluate the efficacy of LADG, we conducted a multi-institutional randomized controlled trial to compare laparoscopy-assisted versus open distal gastrectomy (ODG) for AGC in North China.

**Methods:**

In this RCT, after patients were enrolled according to the eligibility criteria, they were preoperatively assigned to LADG or ODG arm randomly with a 1:1 allocation ratio. The primary endpoint was the morbidity and mortality within 30 postoperative days to evaluate the surgical safety of LADG. The secondary endpoint was 3-year disease-free survival. This trial was registered at ClinicalTrial.gov as NCT02464215.

**Results:**

Between March 2014 and August 2017, a total of 446 patients with cT2-4aN0-3M0 (AJCC 7th staging system) were enrolled. Of these, 222 patients underwent LADG and 220 patients underwent ODG were included in the modified intention-to-treat analysis. The compliance rate of D2 lymph node dissection was identical between the LADG and ODG arms (99.5%, *P* = 1.000). No significant difference was observed regarding the overall postoperative complication rate in two groups (LADG 13.1%, ODG 17.7%, *P* = 0.174). No operation-related death occurred in both arms.

**Conclusions:**

This trial confirmed that LADG performed by credentialed surgeons was safe and feasible for patients with AGC compared with conventional ODG.

Gastric cancer is the fifth most common malignancy, and the third leading cause of cancer death worldwide [1]. Nearly two-thirds of all gastric cancer cases occur in East/Southeast and Central Asia, with 63% cases diagnosed as non-cardia gastric cancer topographically [2]. Because of unhealthy eating habits and infrequent gastroscopic examination, China accounts for 42.6% and 45.0% of the global gastric cancer incidence and mortality, respectively [3]. More than 80% Chinese patients are diagnosed with locally advanced gastric cancer. Among them, almost 60% cases exhibit a single lesion located at the distal stomach [2], thus requiring distal gastrectomy with D2 lymphadenectomy as standard treatment according to the Japanese gastric cancer treatment guidelines [4].

Due to some definite benefits of the minimally invasive surgery, for instance, alleviated pain, shortened hospital stay, and reduced blood loss, laparoscopy-assisted distal gastrectomy has gained growing popularity in China, Japan, and South Korea. Some well-designed multicenter randomized controlled trials (RCTs) from Japan (JCOG0912 [5]) and Korea (KLASS-01 [6]) revealed that LADG was just as safe as conventional ODG in terms of short-term clinical outcomes for stage I gastric cancer, even with significantly lower overall complication rate [6]. Furthermore, the long-term outcomes of EGC patients undergoing LADG were proven comparable to those of patients treated by ODG from a single-arm, multi-institutional clinical trial (JCOG0703 [7]).

Despite the broad application of laparoscopic surgery, whether patients with AGC can benefit from this minimally invasive approach as patients with EGC remains controversial. Owing to several limitations of LADG such as relatively narrow surgical vision, impossible palpation, and more complicated laparoscopic procedures when performing D2 lymph node dissection, operative safety cannot be ensured except skilled surgeons. Besides, the reports of port site seeding and metastases by laparoscopy in the treatment of urinary as well as reproductive system tumors [8–10], and the lack of long-term survival evidence all put the application of LADG for AGC in a challenge.

Around the year 2014, several retrospective studies conducted in East Asia have reported that the application of laparoscopic gastrectomy neither increased surgical risk nor shortened the disease-free survival in AGC patients [11–13]. Further, CLASS-01 in China [14] and JLSSG0901 [15] in Japan have launched nationwide, multi-institutional, two-arm RCTs respectively to compare the safety and efficacy of LADG to ODG for AGC patients. With the wide acceptance of minimally invasive concept, more and more surgeons in North China have completed the learning curve. They found that the fatness degree of patients might influence some detailed surgical procedures when performed LADG. People in North China are more obese than those in the South due to the economic, climate and regional differences [16–18]. According to the epidemiologic survey by He et al., the prevalence of overweight and obesity in rural North China is nearly two-fold as much as that in the South (43.5% vs. 24.8%) [18]. Since obesity is one of the essential factors affecting surgical performance [19, 20], analysis of patients in the northern part of the Chinese mainland will be more instructive for surgeons in this area. Besides, before extensive application of a new surgical technique, the operative safety should be guaranteed first.

Therefore, a multicenter, two-arm, randomized study was launched to investigate the non-inferiority of LADG with D2 lymphadenectomy to ODG in terms of operative safety for locally AGC in North China. And the short-term outcomes are reported herein.

## Patients and methods

### Study design

This study was designed as a multicenter, open-label, non-inferiority, parallel, prospective RCT conducted at five high-volume university hospitals with nine surgeons in Beijing, China. Before initiating this study, the study protocol was approved by the independent ethics committee or institutional review board of each participating institution. This trial was registered with ClinicalTrial.gov (NCT02464215).

The primary endpoint of this clinical trial was the postoperative morbidity and mortality within 30 days. The second endpoint was 3-year disease-free survival. The preoperative staging was evaluated comprehensively by thoracic, abdominal and pelvic computed tomography scans as well as endoscopic ultrasonography of the gastric lesion. All candidates involved in this trial provided written informed consent.

### Patient eligibility

The inclusion criteria for enrolling patients were as follows: Patients age older than 18 years (including 18 years old); Pathologically confirmed primary gastric adenocarcinoma by endoscopic biopsy (including papillary, tubular, mucinous, signet ring cell, and poorly differentiated adenocarcinoma); Tumor located in the lower part of the stomach, potentially resectable by subtotal gastrectomy and D2 lymph node dissection; Preoperative cancer stage cT2-4aN0-3M0 (according to AJCC-7th TNM staging); The Eastern Cooperative Oncology Group performance status of 0 or 1, or the American Society of Anesthesiology classes of I, II, or III; Signed Informed consent.

The exclusion criteria were listed as follows: Surgical history of upper abdomen (except laparoscopic cholecystectomy); Previous gastrectomy, including endoscopic submucosal dissection and endoscopic mucosal resection; Integrated or enlarged lymph node with maximum diameter larger than 3 cm according to preoperative imaging; Other malignant diseases (within 5 years); Preoperative chemotherapy, immunotherapy or radiotherapy; Other illnesses needed operation concurrently; Complications (bleeding, perforation, or obstruction) required emergency surgery due to primary gastric malignancy; Pulmonary function tests FEV1 less than 50% of predicted value; Patient suffered from bleeding tendency disease such as hemophilia or took anti-coagulant medication due to deep vein thrombosis.

### Randomization and masking

As soon as the informed consent was obtained from patients, these eligible patients were assigned to LADG or ODG groups randomly with a 1:1 allocation ratio according to a computer-generated randomization list. The randomization was carried out centrally by the contract research organization (CRO, Beijing High-land Med-Tech Development, Beijing, China), and was not masked for both participating surgeons and patients. The whole process of this trial was monitored by the CRO mentioned above, as well.

### Quality control

All surgeons involved in this trial were specialized in gastric surgery, and have already conducted at least 60 ODG and 60 LADG with D2 lymphadenectomy previously. Each participating institute could perform at least 80 gastrectomies for advanced gastric cancer patients each year. Intraoperative photographs and unedited videos were mandatory required and monitored by the study chair to control the surgical quality. Ten photos were uploaded for each participant. Among them, five pictures were taken for lymph node dissection fields, four for the lesion and resection margins of specimens, and one for the abdominal incision.

### Interventions and outcome measurements

With the exception of surgical approach, all procedures of LADG and ODG group were identical. General anesthesia and tracheal intubation were used during operation in all patients. The location of trocars was not limited while the number should be less than five in the LADG group. After confirmed no metastasis in the abdominal cavity and assessed the resectability of the lesion by laparoscopic exploration, gastrectomy was performed with total omentectomy. The extent of lymphadenectomy adhered principles of Japanese gastric cancer treatment guidelines, as well [4]. Reconstruction was not limited in this trial. Surgeons performed standard Billroth-I (B-I), Billroth-II (B-II), or Roux-en-Y fashion according to their preferences. In the LADG arm, only one mini-laparotomy incision less than 10 cm was permitted.

All the surgical- and medical-related adverse events were documented in detail postoperatively. Wound problems referred to seroma, hematoma, wound infection, and wound dehiscence which occurred on surgery wound and needed additional treatment. Intra-abdominal abscess was confirmed by ultrasonography or tomography, with an increase in WBC and temperature. Anastomotic leakage was defined as gastric contents leaked through drainage tube or proven by upper gastrointestinal tomography. Gastroparesis referred to the obstructed passing of foods through the anastomotic part, with no opinions of intestinal obstruction, stenosis, leakage, or peritonitis. Pancreatic fistula is described as any measurable volume of drain fluid with amylase activity greater than 3 times the upper normal serum value on or after postoperative day 3 [21]. Pulmonary complications were proven by chest X-ray, with symptoms of fever, increased WBC, or even difficulty in breathing. The severity of postoperative morbidity was assessed based on the Clavien–Dindo classification [22].

### Sample size

In order to evaluate the surgical safety of LADG, the overall postoperative complication rate was set as the primary endpoint of this trial. According to the previous reports of Degiuli, the total morbidity rate of standard gastrectomy with D2 lymph node dissection was estimated at 16.3% [23, 24], and the margin of non-inferiority was assumed to be 10%. With a type I error of 0.025 (one-sided) and 80% power, 220 patients were required per group. The sample size was calculated by using PASS11 (NCSS, East Kaysville, UT, USA).

### Statistics

Continuous variables were expressed as the mean ± standard deviation, and categorical variables were presented as frequencies along with percentages. The differences between groups were tested by Student *t* test, *χ*^2^ or Fisher’s exact test where appropriate. The relative risk with 95% confidence interval (CI) for the intraoperative and postoperative complication rates of the LADG group was calculated relative to the reference group. A multivariate binary logistic regression analysis was conducted to determine independent risk factors for postoperative morbidity. All *P* values were two-sided, and *P* values less than 0.05 were considered statistically significant. All statistical analyses were conducted with SPSS (Version 20.0 for Windows; SPSS Inc., Chicago, IL).

## Results

**Fig. 1:**
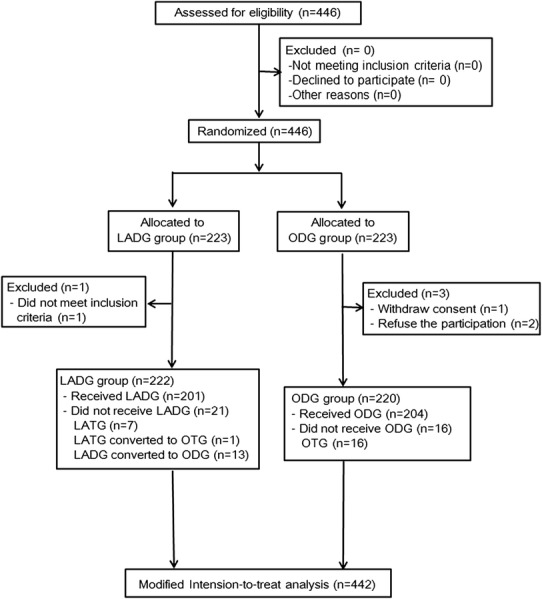
CONSORT diagram

Between March 2014 and August 2017, 446 patients were enrolled from 5 high-volume specialized hospitals in Beijing and were randomly assigned to the LADG or ODG group evenly (*n* = 223 per group). Of these, a total of four patients were excluded after randomization. In the LADG arm, one patient was excluded due to the previous diagnosis of gastric cancer could not be confirmed by endoscopic biopsy at the participating hospital. In the ODG arm, three patients were excluded: one patient withdrew his consent, and other two patients who strongly preferred minimal invasive surgery refused the assigned surgery (Fig. 1). Clinical data of 222 patients in the LADG group and 220 patients in the ODG group were collected thoroughly and entered into an electronic Case Report Form (https://apps.bmclinsys.com/hld-cro/MainFrame.aspx) to analyze surgical outcomes.

The clinical and pathological characteristics of involved patients are listed in Table 1. The mean age was 59.4 years in the LADG arm and 60.6 years in the ODG arm, with the male-to-female ratio of approximately 2:1 in each group. Other baseline factors including BMI, ASA score, comorbidities, tumor location, tumor size, histological type and the pathological stages were all well balanced between these two groups.

**Table 1:** Patient baseline and pathological characteristics

Characteristics	LADG group (*n* = 222)				ODG group (*n* = 220)				*P*
	No.	%	Mean	SD	No.	%	Mean	SD	
Sex									0.338
Male	144	64.9			133	60.5			
Female	78	35.1			87	39.5			
Age (years)			59.4	12.4			60.6	10.2	0.291
BMI (kg/m^2^)			23.1	3.1			23.5	3.3	0.243
ASA score									0.209
I	96	43.2			83	37.7			
II	124	55.9			131	59.5			
III	2	0.9			6	2.7			
No. of comorbidities									0.485
0	152	68.5			157	71.4			
1	47	21.2			50	22.7			
2	18	8.1			10	4.5			
≥ 3	5	2.3			3	1.4			
Comorbidity									
Diabetes	22	9.9			19	8.6			
CVD	16	7.2			8	3.6			
Hypertension	46	20.7			38	17.3			
COPD	0	0			2	0.9			
Hepatic	0	0			1	0.5			
Renal	1	0.5			0	0			
Cerebrovascular	2	0.9			1	0.5			
Others	14	6.3			11	5.0			
Tumor size (cm)			3.6	1.8			3.9	2.2	0.106
Histology									0.164
Differentiated	47	21.2			59	26.8			
Undifferentiated	175	78.8			161	73.2			
Tumor location									0.865
Upper	2	0.9			2	0.9			
Middle	29	13.1			35	15.9			
Lower	181	81.5			173	78.6			
Whole	10	4.5			10	4.5			
Pathological T stage^a^									0.656
T1	58	26.1			52	23.6			
T2	45	20.3			35	15.9			
T3	65	29.3			71	32.3			
T4a	49	22.1			57	25.9			
T4b	5	2.3			5	2.3			
Pathological N stage^a^									0.662
N0	100	45.0			93	42.3			
N1	43	19.4			43	19.5			
N2	30	13.5			39	17.7			
N3	49	22.1			45	20.5			
Pathological M stage^a^									0.543
M0	218	98.2			214	97.3			
M1	4	1.8			6	2.7			
Pathological TNM stage^a^									0.982
IA	44	19.8			41	18.6			
IB	31	14.0			27	12.3			
IIA	31	14.0			31	14.1			
IIB	32	14.4			32	14.5			
IIIA	32	14.4			28	12.7			
IIIB	28	12.6			32	14.5			
IIIC	20	9.0			23	10.5			
IV	4	1.8			6	2.7			

*LADG* laparoscopy-assisted distal gastrectomy, *ODG* open distal gastrectomy, *SD* standard deviation, *BMI* body mass index, *ASA* American society of anesthesiologists, *CVD* cardiovascular disease except hypertension, *COPD* chronic obstructive pulmonary disease

^a^Pathologic stage according to the American Joint Committee on Cancer, 7th Edition

The surgical outcomes are summarized in Table 2. Distal gastrectomy was carried out for 214 patients (96.4%) in the LADG group and 204 patients (92.7%) in the ODG group (*P* = 0.089). About 99.5% participants underwent D2 lymphadenectomy in the LADG group, so did patients in the ODG group (*P* = 1.000). The reconstruction methods performed by surgeons were comparably distributed in each group, as well (*P* = 0.149). With respect to the mean surgical time, the LADG group took 32.6 min longer than the ODG group (242.5 ± 63.5 vs. 209.9 ± 53.6, *P* < 0.001). And the estimated blood loss in the LADG group was 26.1 mL less than the blood loss in the ODG group (91.4 ± 90.9 vs. 117.5 ± 103.5, *P* = 0.005). During operation, 14 patients in the LADG group underwent open conversion because of locally advanced tumors which invaded surrounding organs (*n* = 6), uncontrolled bleeding (*n* = 2), dense adhesions blurred the surgical field (*n* = 4), and length of incision more than 10 cm (*n* = 2). Concerning the recovery process, patients in the LADG group got a shorter duration to first flatus (*P* = 0.013), a much earlier first liquid intake (*P* = 0.003), and a less postoperative hospital stay (*P* = 0.018) compared with patients in the ODG group. There was no significant difference with regard to intraoperative blood transfusion rate (*P* = 0.984), the length to the proximal (*P* = 0.169) or distal (*P* = 0.501) resection margin, time to ambulation (*P* = 0.274), and the retrieved lymph nodes (*P* = 0.083) between the two arms.

**Table 2:** Surgical outcomes of the LADG and ODG groups

Outcomes	LADG group (*n* = 222)				ODG group (*n* = 220)				*P* ^a^
	No.	%	Mean	SD	No.	%	Mean	SD	
Gastrectomy									0.089^b^
Distal	214	96.4			204	92.7			
Total	8	3.6			16	7.3			
Reconstruction									0.149^b^
Billroth-I	97	43.7			90	40.9			
Billroth-II	105	47.3			97	44.1			
Roux-en-Y	19	8.6			33	15.0			
Others	1	0.5			0	0			
Lymphadenectomy									1.000^c^
D2	221	99.5			219	99.5			
Others	1	0.5			1	0.5			
Combined resection	1	0.5			4	1.8			0.215^c^
Partial liver	0	0			2	0.9			
Spleen	0	0			1	0.5			
Partial transverse colon or mesentery	1	0.5			1	0.5			
Surgical time (min)			242.5	63.5			209.9	53.6	0.000
Estimated blood loss (mL)			91.4	90.9			117.5	103.5	0.005
Open conversion	14	6.3							
Locally advanced tumor (T4 stage)	6								
Uncontrolled bleeding	2								
Adhesion	4								
Length of incision > 10 cm	2								
Intraoperative blood transfusion	9	4.1			9	4.1			0.984^b^
Proximal resection margin (cm)			5.0	2.2			5.3	2.5	
Distal resection margin (cm)			3.8	2.4			3.9	2.7	0.501
Retrieved lymph nodes			29.5	10.4			31.4	12.3	0.083
Length of incision (cm)			8.1	3.7			16.7	3.8	0.000
Time to ambulation (days)			1.2	1.0			1.4	1.8	0.274
Time to first flatus (days)			2.8	1.0			3.1	1.4	0.013
Time to first liquid intake (days)			7.0	1.8			7.9	3.7	0.003
Postoperative hospital stay (days)			9.9	3.7			10.9	5.2	0.018

*LADG* laparoscopy-assisted distal gastrectomy, *ODG* open distal gastrectomy, *SD* standard deviation

^a^Student *t* test

^b^Pearson Chi-square test

^c^Fisher’s exact method

Surgery-related complications are presented in Table 3. Intraoperative adverse events happened in five patients (2.3%) in the LADG group (more than 400 mL bleeding in three patients, spleen injury in one patient and diaphragm injury in one patient), and six patients (2.7%) in the ODG group (more than 400 mL bleeding in five patients and transfusion allergic reaction in one patient). In terms of overall postoperative morbidity within 30 days, no marked significance was observed between the LADG and ODG arms (13.1% vs. 17.7%, *P* = 0.174). Besides, each subtype of complication was distributed similarly in the two groups. No intraoperative or postoperative death happened in either group. The leading causes of postoperative adverse events were composed of the pulmonary problem, gastroparesis, and anastomotic leakage. Moreover, four patients in the LADG group and eight patients in the ODG group underwent reoperation due to anastomotic leakage (three vs. four), intestinal fistula (one vs. zero), intraluminal bleeding (zero vs. one), intra-abdominal bleeding (zero vs. two), and pancreatic fistula (zero vs. one).

**Table 3:** Morbidity and mortality in the LADG and ODG groups within 30 postoperative days

Morbidity type	LADG group (*n* = 222)		ODG group (*n* = 220)		*P* ^a^	RR (95% CI)
	No.	%	No.	%		
Intraoperative complication	5	2.3	6	2.7	0.749^b^	0.826 (0.256–2.666)
Postoperative complication	29	13.1	39	17.7	0.174^b^	0.737 (0.473–1.148)
Wound problem	3	1.4	2	0.9	1.000	1.486 (0.251–8.810)
Fluid collection/abscess	2	0.9	4	1.8	0.448	0.495(0.092–2.678)
Intra-abdominal bleeding	0	0	2	0.9	0.247	
Intraluminal bleeding	1	0.5	2	0.9	0.622	0.495 (0.045–5.425)
Ileus	1	0.5	2	0.9	0.622	0.495 (0.045–5.425)
Intestinal fistula	1	0.5	0	0	1.000	
Anastomotic leakage	3	1.4	4	1.8	0.723	0.743 (0.168–3.282)
Lymphatic leakage	2	0.9	3	1.4	0.685	0.661 (0.111–3.915)
Gastroparesis	6	2.7	10	4.5	0.322	0.595 (0.220–1.608)
Pancreatic fistula	0	0	1	0.5	0.498	
Pulmonary problem	10	4.5	8	3.6	0.811	1.239 (0.498–3.080)
Renal problem	0	0	1	0.5	0.498	
Mortality	0	0	0	0		
Clavien–Dindo classification						0.780^b^
I	3	1.4	2	0.9	1.000	1.486 (0.251–8.810)
II	21	9.5	28	12.7	0.292	0.743 (0.436–1.268)
IIIa	1	0.5	1	0.5	1.000	0.991 (0.062–15.744)
IIIb	4	1.8	8	3.6	0.259	0.495 (0.151–1.622)
IV	0	0	0	0		

*LADG* laparoscopy-assisted distal gastrectomy, *ODG* open distal gastrectomy

^a^Fisher’s exact method

^b^Pearson Chi-square test

Furthermore, univariate and multivariate analyses were conducted to distinguish risk factors which may influence the occurrence of postoperative morbidity. As shown in Table 4, the operative approach, number of comorbidities, type of reconstruction, operative time, and the pathological stage had no significant effect on the development of postoperative complications. Meanwhile, the age (OR ≥ 60 2.362, 95% CI 1.236–4.512; *P* = 0.009) and BMI (OR ≥ 25 2.013, 95% CI 1.101–3.680, *P* = 0.023) were identified as independent risk factors for postoperative complications through multivariate analysis.

**Table 4:** Univariate and multivariate analyses of risk factors for postoperative complications

Variables	No. (*n* = 442)	Univariate analysis		Multivariate analysis	
		Morbidity (*n* = 68)	*P*	Odds ratio^a^	*P*
Operative approach			0.174		
Laparoscopy	222	29 (13.1%)			
Open	220	39 (17.7%)			
Age (years)			0.003		
< 60	189	18 (9.5%)		1	0.009
≥ 60	253	50 (19.8%)		2.362 (1.236–4.512)	
Sex			0.142		
Male	277	48 (17.3%)			
Female	165	20 (12.1%)			
BMI (kg/m^2^)			0.025		
< 25	322	42 (13.0%)		1	0.023
≥ 25	120	26 (21.7%)		2.013 (1.101–3.680)	
No. of comorbidities			0.350		
0	309	42 (13.6%)			
1	97	18 (18.6%)			
2	28	7 (25.0%)			
≥ 3	8	1 (12.5%)			
Reconstruction type			0.114		
Billroth-I	187	22 (11.8%)			
Billroth-II	202	34 (16.8%)			
Roux-en-Y	53	12 (22.6%)			
Operative time (min)			0.322		
≤ 200	180	24 (13.3%)			
> 200	262	44 (16.8%)			
Pathological T stage^b^			0.707		
T1	110	15 (13.6%)			
T2	80	10 (12.5%)			
T3	136	24 (17.6%)			
T4	116	19 (16.4%)			
Pathological N stage^b^			0.075		
N0	193	23 (11.9%)			
N1-3	249	45 (18.1%)			
Pathological TNM stage^b^			0.131		
I	143	17 (11.9%)			
II	126	16 (12.7%)			
III	163	32 (19.6%)			
IV	10	3 (30%)			

*BMI* body mass index

^a^The 95% confidence interval was given in parentheses

^b^Pathologic stage according to the American Joint Committee on Cancer, 7th Edition

We further analyzed the association between BMI and surgical complications in each group, and the results are listed in Table 5. The proportion of postoperative morbidity was lower in the subset of BMI < 25, compared with the subgroup of BMI ≥ 25 in the LADG group (9.3% vs. 23.3%, *P* = 0.006). While in the ODG group, the rate of postoperative morbidity was similar no matter whether BMI < 25 or ≥ 25 (16.9% vs. 20.0%, *P* = 0.589).

**Table 5:** The analysis of BMI and postoperative morbidity in the LADG or ODG group

BMI (kg/m^2^)	LADG group		*P*	ODG group		*P*
	No.(*n* = 222)	Morbidity (*n* = 29)		No. (*n* = 220)	Morbidity (*n* = 39)	
< 25	162	15 (9.3%)	0.006	160	27 (16.9%)	0.589
≥ 25	60	14 (23.3%)		60	12 (20.0%)	

*LADG* laparoscopy-assisted distal gastrectomy, *ODG* open distal gastrectomy, *BMI* body mass index

## Discussion

With the acceptance of minimally invasive concept, whether gastric cancer patients could benefit from the application of laparoscopy has attracted more and more attention. As LADG is upgraded from an investigational treatment to an optional therapy for clinical stage I GC after years of practice [25], various studies have concerned the feasibility and safety of LADG for patients with AGC. A majority of research compared LADG with ODG were retrospective studies [11–13]. What is more, conclusions from retrospective or prospective studies even meta-analyses [26, 27] concerning this topic were inconsistent. Some literature concluded that LADG was associated with a lower postoperative complication rate in AGC patients [26, 27]; others found no significant difference between LADG and ODG concerning postoperative morbidity [14, 28]. Even a meta-analysis reported by Cochrane Database considered that the evidence qualities of most completed or ongoing RCTs were low because of high risks of bias [29]. Since operative safety is an essential prerequisite and basis for new surgical procedures, well-designed trials are needed to validate the safety and efficacy of LADG for AGC patients.

The present RCT, which recruited patients from North China with unrestricted BMI and upper age limit, therefore partially represented the real-world data, showed no remarkable difference between LADG and ODG regarding postoperative morbidity. The well-known advantages of LADG reported in EGC patients [5, 6], such as less blood loss and faster postoperative recovery was confirmed in this trial again. Due to the magnified surgical field and more meticulous surgical procedures, unexpected bleeding and excessive distraction could be prevented efficiently. Patients in both arms were forced to resume off-bed activities the day after the operation, which contributed to the similar time to ambulation shown in Table 2.

The overall postoperative complication rate in our study was comparable to the morbidity reported by the nationwide CLASS-01 trial in China [14]. Pulmonary complications accounted for the most considerable proportion of postoperative adverse events. Advanced age, cardiac and pulmonary comorbidities, reduced diaphragmatic activity due to incisional pain and microatelectasis were possible reasons led to such complications [30, 31].

Different from CLASS-01, gastroparesis was also another leading cause of postoperative adverse events. The incidence of gastroparesis in this trial was comparable to the previous reports of 0.4–7% [6, 14, 32–34]. The etiology of postsurgical gastroparesis syndrome (PGS) was diverse. Recent studies identified several risk factors for PGS, such as patient BMI ≥ 25 kg/m^2^, age ≥ 65 years, B-II reconstruction and the like [33, 35]. In the present study, PGS occurred in 16 patients in total. Among them, the BMI of eight patients was higher than 25 kg/m^2^, with three of them even higher than 30 kg/m^2^ (BMI < 25 vs. ≥ 25, 2.5% vs. 6.7%, *P* = 0.046); the age of seven patients was older than 65 years (Age < 65 vs. ≥ 65, 3.1% vs. 4.5%, *P* = 0.446); and eight of them received B-II construction (B-II vs. non-B-II, 4.0% vs. 3.3%, *P* = 0.725). Although the number of patients with PGS was not enough for us to draw definite conclusions, obesity partially contributed to the incidence of PGS in our study.

As for the conversion rate from LADG to ODG, the proportion present in this trial was similar to that reported in CLASS-01 (6.4%) [14], but was higher than that seen in KLASS-01 (3.2%) [6], JCOG0912 (3.5%) [5], and JCOG0703 (2.9%) [36]. One potential reason might be the locally advanced tumors (T4) which invaded surrounding organs and were difficult to handle through laparoscopy in AGC patients. Besides, surgeons intended to achieve the same surgical quality as obtained from ODG for the sake of patient’s benefit.

Similar as the results from CLASS-01 and KLASS-01 trials [6, 14], the incidences of pancreatic fistula and the severe morbidity rate which referred to postoperative complications no less than grade IIIa according to the Clavien-Dindo classification [22] were both relatively low in our study. The definition of postoperative pancreatic fistula (POPF) which we followed in the present trial has been updated in 2016 by the International Study Group of Pancreatic Fistula (ISGPS) [21]. Compared with the definition of POPF in 2005 [37], increased amylase activity without clinical impact was defined as biochemical leak rather than pancreatic fistula. Besides, operations performed by credentialed surgeons were much more meticulously. Therefore, less complications of pancreatic fistula were recorded in our study. The Clavien–Dindo classification defines the severity of surgical complications according to the type of corresponding therapy [22]. If the treatment of postoperative morbidity does not require surgical, endoscopic or radiological intervention, the severity classification of postoperative morbidity will be less than grade III [22]. Take anastomotic leakage for example, if the patient needs reoperation, the Clavien–Dindo grade is IIIb; if the subject needs percutaneous drainage treatment, the grade is IIIa. While if only long-term total parenteral nutrition and fasting can alleviate this complication, it will be leveled as grade II. As Lee et al. described, one of the most important risk factors for laparoscopic gastrectomy is the experience of the surgeon [38]. All participated surgeons in our study have completed the learning curve and were full of experience. Furthermore, with the continuous improvement of perioperative nursing care and the practice guided by the concept of Enhanced Recovery After Surgery (ERAS), severe surgical complications are getting less and less.

Moreover, the mean retrieved lymph nodes were comparable in two arms in our study (LADG vs. ODG: 29.5 ± 10.4 vs. 31.4 ± 12.3, *P* = 0.083), which was in the range of 23.0–43.3 as reported previously [6, 39, 40], but slightly less than that presented in CLASS-01 [14]. The number of harvested lymph nodes was not only associated with the surgical technique but also correlated with the thoroughness of pathological examination, state of specimens and the innate number of lymph nodes for each patient [39]. In China, experienced surgeons after learning curve could harvest 26.2–28.8 LNs in D2 lymphadenectomy [41, 42]. Although some recent studies suggested that higher retrieved lymph nodes could lead to better prognosis in gastric cancer patients [43], Lu indicated that still at least 16 removed lymph nodes could yield improved prognosis of patients underwent distal gastrectomy [39]. As a multicenter trial, we considered that the retrieved lymph nodes were sufficient in our study.

Because of increased life expectancy and a better quality of life, the number of gastric cancer patients with advanced age and higher BMI is continuously rising, especially in the north part of China [44]. Previous reports indicated that elder age and high BMI contributed to increased morbidity after gastrectomy [45–47]. Similarly, gastric cancer patients who were more than 60 years or BMI higher than 25 kg/m^2^ had a greater risk of postoperative complications in this trial. Regarding the effect of BMI on the surgical results of LADG, we found that higher BMI was a risk factor for the occurrence of complications in the LADG group but not in the ODG group. Compared with ODG, performing LADG in AGC patients with higher BMI is much more difficult due to insufficient visualization of the abdominal cavity. As reported by Noshiro et al. [47], in order to improve the visualization field in fatty patients, surgeons have to use higher insufflation pressure or rotate the operative table extremely when performing LADG, which may cause increased accidental injuries during operation. In addition, because it is harder to isolate blood vessels or dissect lymph nodes which are surrounded by a massive bulk of fat tissues under laparoscopy, the prolonged surgical time and anesthesia duration may lead to more postoperative complications in obese patients. Currently, several studies including ours believed that the proportion of postoperative adverse events might be increased with higher BMI when conducting LADG [47, 48], while a few other groups considered that increased BMI could not raise the likelihood of postoperative morbidity yet [49, 50]. Therefore, whether or not increased BMI influences the surgical results of patients undergoing LADG requires more substantial evidence to draw conclusions.

There were several limitations in the present study. First, few hospitals from other provinces in North China had participated in this multicenter clinical trial. Second, selection bias might existed since the randomization of surgical process was not done in the operating room. At last, besides BMI, a more sophisticated method such as visceral fat area should be applied to evaluate obesity, and thus more accurate results could be obtained to reveal the effect of obesity on the surgical results of LADG. Based on the results of the present study, we are planning to perform a randomized phase-III trial to compare the oncologic efficacy between LADG and ODG, and designs aimed at investigating the effect of obesity are included, as well.

In summary, the results of this RCT demonstrated that LADG performed by credentialed surgeons was surgically safe and feasible for AGC patients compared with conventional ODG. Nevertheless, before the non-inferiority of LADG with regard to oncologic efficacy is generally confirmed, laparoscopic surgery is still an investigational treatment for AGC [25], which needs to be carefully performed under well-established principles.
